# Association between retinal hemorrhagic pattern and macular perfusion status in eyes with acute branch retinal vein occlusion

**DOI:** 10.1038/srep28554

**Published:** 2016-06-23

**Authors:** Yuki Muraoka, Akihito Uji, Akitaka Tsujikawa, Tomoaki Murakami, Sotaro Ooto, Kiyoshi Suzuma, Ayako Takahashi, Yuto Iida, Yuko Miwa, Masayuki Hata, Nagahisa Yoshimura

**Affiliations:** 1 Department of Ophthalmology and Visual Sciences, Kyoto University Graduate School of Medicine, Kyoto, Japan; 2 Department of Ophthalmology, Kagawa University Faculty of Medicine, Miki, Japan

## Abstract

This prospective study included 63 eyes with acute branch retinal vein occlusion (BRVO) to evaluate the retinal hemorrhagic patterns at the posterior poles and explore their clinical relevance in macular perfusion differentiation. Retinal hemorrhagic patterns and macular perfusion status were evaluated via fundus photography and fluorescein angiography, respectively. Macular perfusion was judged as nonischemic in 30, ischemic in 28, and undeterminable in 5 among the 63 eyes. Predominant hemorrhagic patterns were flame-shaped in 39 (67.2%) and non-flame-shaped in 19 (32.8%) eyes. All 39 eyes with a flame-shaped hemorrhage showed a nonischemic macula. Of the 19 eyes classified as having a non-flame-shaped hemorrhage, 13 (68.4%) had an ischemic macula and 6 (31.6%) had a nonischemic macula (*P* < 0.001). Parallelism in eyes with a flame-shaped hemorrhage was higher than in those with a non-flame-shaped hemorrhage (*P* < 0.001), and in those with a nonischemic macula versus those with an ischemic macula (*P* < 0.001). The area under the curve for parallelism was 0.975 (*P* < 0.001), suggesting an accurate diagnostic parameter for macular perfusion differentiation. In conclusion, we objectively evaluated retinal hemorrhagic patterns at the posterior pole in BRVO using the parallelism method, which was useful in differentiating macular perfusion status.

The visual prognosis of branch retinal vein occlusion (BRVO) has been substantially improved since anti-vascular endothelial growth factor (VEGF) agents were introduced^1^,^2^,^3^,^4^. However, despite the use of aggressive anti-VEGF treatments, we still encounter cases with inadequate recovery of visual functions in which retinal ischemia occasionally involves the macular area. Previously, Ota and associates reported that macular function measured by microperimetry was markedly decreased in macular nonperfused areas (NPAs) of eyes with BRVO^5^, although Campochiaro and associates recently reported that anti-VEGF treatments could prevent NPA enlargement and promote reperfusion^6^. In the management of BRVO, it is thus worthwhile to evaluate macular perfusion status in the acute phase.

Fluorescein angiography (FA) is still an essential tool to detect morphologic and functional changes of the retinal vasculature. However, FA requires intravenous injections of dye, and is therefore too invasive to be performed readily^7^,^8^,^9^,^10^,^11^; the development of alternative techniques is thus sought. Some investigators reported that red-free imaging^12^, or angiography^13^ and en-face^14^,^15^,^16^ optical coherence tomography (OCT) images were useful to detect NPAs in eyes with retinal vascular diseases. However, the interpretations and analysis of these images are often complicated, and parts of these modalities are commonly unavailable^13^,^14^,^15^,^16^. In addition, color fundus photography, the most common imaging modality, has not been applied to the analysis of NPAs.

The posterior poles of eyes with acute BRVO exhibit simultaneous retinal ischemia and retinal hemorrhages^17^,^18^. A characteristic feature of the retinal hemorrhages is that they are often referred to as being flame-shaped (fan-shaped or splinter-shaped), and occasionally as a blot or dot (non-flame-shaped)^17^,^18^. Histologically, hemorrhages in the retinal nerve fiber layer are linear because the blood is aligned parallel to the axons, and rounded dot and blot hemorrhages lie in the nuclear and plexiform layers^19^. However, no information is available on associations between retinal hemorrhagic pattern and perfusion status; a reason may be because it is difficult to evaluate hemorrhagic patterns objectively.

In the current study, we included consecutive patients with acute BRVO and evaluated retinal hemorrhagic patterns at the posterior pole not only qualitatively, but also quantitatively, and explored their clinical relevance in the differentiation of macular perfusion status.

## Results

Table 1 shows the measurement values from all patients who were eligible for inclusion in the current study. On examination, all eyes showed visual disturbance due to acute BRVO; mean visual acuity was 0.42 ± 0.36, and the mean symptom duration before examination was 1.7 ± 1.3 months. Retinal perfusion status at the posterior pole was judged as nonischemic in 30 eyes, ischemic in 28 eyes, and undeterminable in 5 eyes (2 eyes with macular BRVO and 3 eyes with major BRVO) because of blockage by a dense retinal hemorrhage (Table 1). These 5 eyes, whose retinal perfusion status at the posterior pole was undeterminable, were excluded from the analysis in the current study.

### Hemorrhagic patterns of eyes with acute BRVO

**Figure 1:**
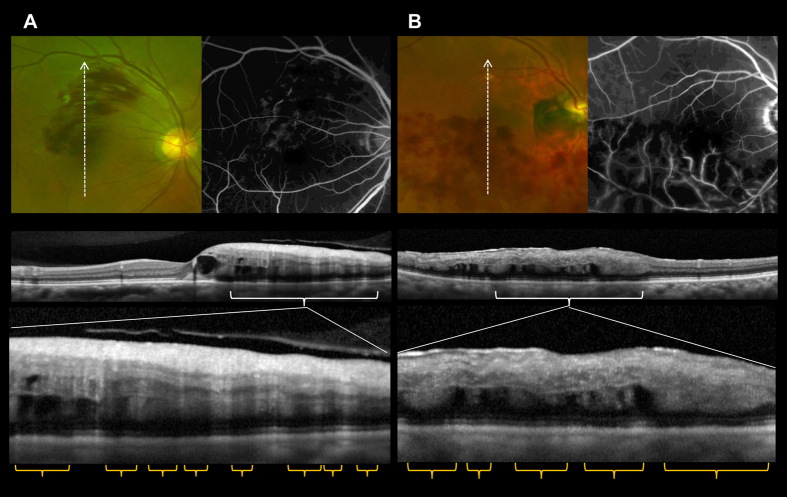
Optical coherence tomography (OCT) of macular areas of eyes with acute branch retinal vein occlusion. (**A,B**) Color fundus photography, fluorescein angiography (FA), and OCT sections of a macular area with a typical flame-shaped hemorrhage, and those of a non-flame-shaped blot hemorrhage, respectively. (**A**) FA shows a nonischemic macula. OCT sections reveal an organized layered structure in the inner retina, and the locations of flame-shaped retinal hemorrhages are consistent with moderately reflective cylindrical bands that block visualization of the outer retina. (**B**) FA shows an ischemic macula. OCT sections show that the layered structures of the inner retina are disorganized, the boundaries between inner retinal layers are obscured, and the retinal hemorrhages correspond to amorphous and moderately hyperreflective lesions located not at the inner retina, but at the outer retina.

**Figure 2:**
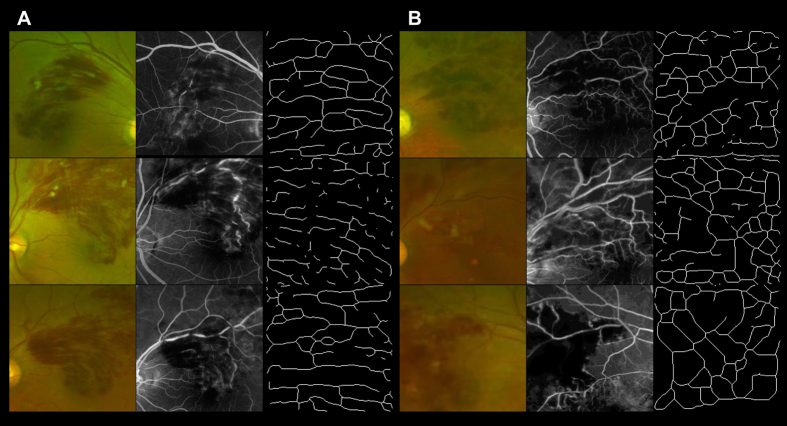
The association between retinal hemorrhagic pattern and perfusion status at the posterior poles of eyes with acute branch retinal vein occlusion. (**A,B**) Optos color fundus photography, fluorescein angiography (FA), and skeletonized images reflecting parallelism of the posterior poles of eyes exhibiting a flame-shaped retinal hemorrhage and non-flame-shaped retinal hemorrhage, respectively. Detailed comparisons between color fundus photography and FA at the posterior pole reveal that macular areas with flame-shaped hemorrhages are nonischemic (**A**); however, macular areas with non-flame-shaped hemorrhages are often accompanied by retinal nonperfusion (**B**).

The 58 eyes included in the analysis exhibited retinal hemorrhages in the areas affected by acute BRVO, some of which were accompanied by cotton wool spots (CWS), retinal whitening, and sheathed retinal vessels (Table 2). In the peripheral retina, no hemorrhage was observed in eyes with macular BRVO. In eyes with major BRVO or hemi-CRVO, non-flame-shaped (dot or blot) hemorrhages were predominant in peripheral areas. At the posterior pole, however, a flame-shaped (and sometimes fan-shaped or splinter-shaped) retinal hemorrhage was also observed in addition to a non-flame-shaped hemorrhage (Figs 1 and 2). We qualitatively determined the hemorrhagic patterns at the posterior pole in a blinded fashion, which were flame-shaped in 39 (67.2%) eyes and non-flame-shaped in 19 (32.8%) eyes.

In all eyes with a flame-shaped hemorrhage in the macular area, OCT revealed an organized layered structure of the inner retina (Fig. 1). In OCT sections of eyes with a non-flame-shaped hemorrhage in the macular area, however, the layered structures of the inner retina were disorganized and the boundaries between inner retinal layers were obscured in 18 (94.7%) eyes. The retinal hemorrhages corresponded to amorphous and moderate hyper-reflective lesions located not at the inner retina, but at the outer retina, and often extended to the outer plexiform layer (Fig. 1).

### Association between retinal hemorrhagic patterns and perfusion status at the posterior pole

Detailed comparisons between color fundus photography and FA at the posterior pole revealed that all 39 eyes classified as having a flame-shaped hemorrhage at the posterior pole showed a nonischemic macula, while 13 (68.4%) of the 19 eyes classified as having a non-flame-shaped hemorrhage had an ischemic macula and 6 (31.6%) had a nonischemic macula (*P* < 0.001, Table 3, Fig. 2).

### Parallelism of retinal hemorrhagic patterns and perfusion status at the posterior pole

**Figure 3:**
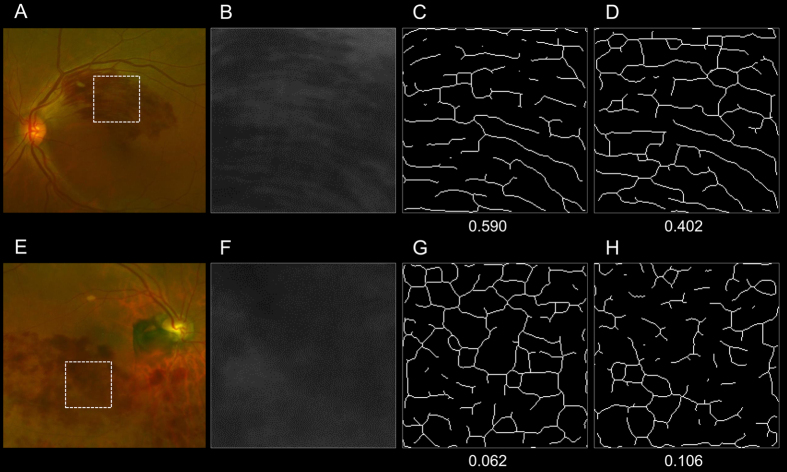
Quantification of retinal hemorrhagic patterns at the posterior pole of eyes with acute branch retinal vein occlusion using the parallelism method. Images of the posterior pole of an eye with a typical flame-shaped retinal hemorrhage (**A–D**) and of an eye with a non-flame-shaped blot retinal hemorrhage (**E–H**) are shown. Optos color fundus photographs (**A,E**), the split green channel of the originals (**B,F**), extracted skeletonized images of the retinal hemorrhage (**C,G**), and hemorrhage-sparse areas (**D,H**) are shown. The skeletonized images are made by applying a filter using ImageJ software followed by binarization with Otsu’s thresholding method. The parallelism values are then calculated based on the processed images. Parallelism is expected to show higher values in eyes with a flame-shaped retinal hemorrhage and lower values in eyes with a non-flame-shaped dot or blot retinal hemorrhage. The averaged parallelism values are 0.496 [(0.590 + 0.402)/2] in an eye with a flame-shaped retinal hemorrhage (**C,D**) and 0.084 [(0.062 + 0.106)/2)] in an eye with a non-flame-shaped blot retinal hemorrhage (**G,H**).

We applied the method of parallelism for more objective and quantitative evaluations of retinal hemorrhagic patterns at the posterior pole (Fig. 3). The parallelism values effectively reflected hemorrhagic patterns associated with acute BRVO. Parallelism in eyes classified as having flame-shaped hemorrhages at the posterior pole was significantly higher than in eyes with non-flame-shaped hemorrhages (0.333 ± 0.110 vs. 0.122 ± 0.067, *P* < 0.001, Fig. 3).

Table 3 shows comparisons of clinical measurements of eyes with acute BRVO divided by the perfusion status at the posterior pole. The parallelism values of eyes with a nonischemic macula were significantly higher than those of eyes with an ischemic macula (0.362 ± 0.115 vs. 0.222 ± 0.126, *P* < 0.001, Table 3). Other quantitative measurements including age, symptom duration, visual acuity, and mean foveal thickness were not different depending on perfusion status at the posterior pole (*P* = 0.693, 0.294, 0.345, and 0.169, respectively; Table 3).

Finally, to investigate how correctly the continuous variables differentiated macular perfusion status, we calculated AUROCs, the best sensitivity-specificity balance, and sensitivities at 90% and 95% specificities for age, symptom duration, visual acuity, mean foveal thickness, and retinal hemorrhagic pattern (parallelism) at the posterior pole (Table 4). The AUROC was 0.975 (*P* < 0.001) for parallelism values (quantitative measurements of hemorrhagic patterns), which were significantly accurate diagnostic parameters (Table 4).

## Discussion

One of the major complications associated with BRVO is macular ischemia^5^,^20^, which causes upregulations of VEGF, hyperpermeability of the affected capillaries, and resultant macular edema. Although Finkelstein reported that the visual prognoses of eyes with incomplete macular perfusion were better than those with complete macular perfusion in a longitudinal observation of BRVO cases^20^, macular ischemia can cause severe visual impairment during the acute phase because the affected inner retina fails to carry out phototransduction^21^. Therefore, clinicians should always be aware of the macular perfusion status of the patient.

Although FA is still an essential tool for evaluations of retinal circulatory status, FA is inconvenient and unable to be performed easily or multiple times because of its invasive nature^7^,^8^,^9^,^10^,^11^. Instead of FA, en-face images of swept-source OCT (SSOCT) were reported to visualize NPAs^14^,^15^,^16^, retinal thinning as an indicator of an NPA^14^, and honeycomb-like structures^16^ in a case report and small BRVO case series. Shin and associates reported that confocal red-free imaging could detect NPAs as well as FA in eyes with BRVO^12^. Ruminski and associates most recently reported 3-dimensional morphologic changes in the superficial and deep capillary plexuses of eyes with BRVO using OCT angiography^13^. However, these studies did not include eyes with acute BRVO, presumably because image quality became impaired due to the presence of a fresh retinal hemorrhage^12^,^13^,^14^,^16^. In addition, most modalities are commonly unavailable^13^,^14^,^15^,^16^.

The posterior poles of eyes with acute BRVO exhibit simultaneous retinal ischemia and retinal hemorrhages. A characteristic feature of the retinal hemorrhages is that they are often referred to as flame-shaped (fan-shaped or splinter-shaped), and occasionally as a blot or dot (non-flame-shaped)^17^,^18^. In the current study, detailed comparisons between color fundus photography and FA at the posterior pole revealed that a flame-shaped hemorrhage was associated with a nonischemic macula; however, dot and blot (non-flame-shaped) hemorrhages were often accompanied by an ischemic macula. However, this result would be biased by subjective evaluations of hemorrhagic pattern. We therefore made an attempt to apply parallelism for a more objective and quantitative analysis.

Parallelism was originally developed to evaluate intracellular motility such as organelle trafficking and streaming of the endoplasmic reticulum in plants^22^,^23^. Recently, Uji and associates successfully applied a quantitative evaluation to the foveal pathomorphology of eyes with an epiretinal membrane^24^ or diabetic macular edema^25^ using OCT imaging. In the present study, parallelism was used in the analysis of hemorrhagic patterns on color fundus photography. In eyes with higher parallelism values, components of the retinal hemorrhages were most parallel to each other, which were typically shown in flame-shaped hemorrhages. In contrast, in eyes with lower parallelism values, components of the retinal hemorrhage were less parallel, which were not shown as flame-shaped, but as non-flame-shaped, rounded-hemorrhages. The parallelism in eyes with flame-shaped hemorrhages was significantly higher than in eyes with non-flame-shaped hemorrhages (0.333 ± 0.110 vs. 0.122 ± 0.067). In addition, parallelism values in eyes with a nonischemic macula were significantly higher than in those with an ischemic macula (0.362 ± 0.115 vs. 0.222 ± 0.126), which both effectively reflected the hemorrhagic pattern and differed depending on perfusion status at the posterior pole.

In pathology of the human retina, hemorrhages in the retinal nerve fiber layer are linear because the blood is aligned parallel to the axons, and rounded, dot-, and blot-shaped hemorrhages lie in the nuclear and plexiform layers^19^. In an ischemic retina, some retinal nerve fibers are swollen because of stagnant axonal flow and others are disorganized depending on nonperfusion severity^26^. The strong correlations observed in the current study between flame-shaped hemorrhages and nonischemic retinas, and between non-flame-shaped hemorrhages and ischemic retinas may partially result from the association of perfusion status with pathomorphological changes of the retinal nerve fiber layer. In a nonischemic retina, the retinal nerve fibers are kept in a bundle shape; however, they are morphologically disorganized in an ischemic retina. We herein speculate that extravasated red blood cells in the retinal nerve fiber layers might play a role in the so-called “stain” accentuating retinal nerve fiber appearance. In the peripheral retinas of the included eyes, non-flame-shaped (dot and blot) hemorrhages were predominant. Previous histological studies reported that the inner retina was thinner depending on the distance from the optic disc^27^, and that a peripheral blot hemorrhage was limited by undamaged neurons or Müller cells^19^. This might be a reason why flame-shaped hemorrhage was uncommon in the peripheral retina, even when nonischemic.

Imai and associates reported that en-face SSOCT images could detect thinning of the inner retinal layer as an indicator of NPAs in eyes with BRVO^14^, and Dodo and associates reported that NPAs in diabetic retinopathy were associated with the absence of a boundary between the retinal nerve fiber layer and ganglion cell layer or inner plexiform layer on spectral domain OCT images (SDOCT)^28^. In the current study, SDOCT sections of a macular area with a non-flame-shaped hemorrhage showed that layered structures of the inner retina were disorganized and boundaries between the inner retinal layers were obscured in most eyes (94.7%), which is consistent with the previous OCT studies. In addition, on the SDOCT sections, non-flame-shaped retinal hemorrhages were detected corresponding to amorphous and moderate hyper-reflective lesions located not at the inner retina, but at the outer retina, and often extended to the outer plexiform layer, which was similar to previously reported histological findings^19^. Thinning of the inner retina in an NPA might push the hemorrhage toward the outer retina. As another explanation, a hemorrhage from the outer capillary plexus in a case with an ischemic macula might be superior to that with a nonischemic macula, and the retinal hemorrhage would subsequently tend to locate at the outer retina.

As ocular features associated with retinal perfusion, the prevalence of CWS, retinal whitening, and sheathed vessels were also examined using Optos color fundus imaging. However, the presence or absence of these retinal findings was not significantly different depending on perfusion status at the posterior pole in acute BRVO. The small number of included patients and lower prevalence of CWS (31.0%), retinal whitening (3.4%), and sheathed vessels (10.3%) would cause the observed weak associations with perfusion status. In contrast, a higher prevalence (presumably 100%) of retinal hemorrhage may contribute to a strong association between hemorrhagic pattern and retinal perfusion status.

The major limitations of our study are its small sample size and cross-sectional nature. While retinal perfusion status and retinal hemorrhages are known to transmute in eyes with retinal vein occlusion, these longitudinal changes were not examined. In the chronic phase of BRVO in which most retinal hemorrhages resolve, hemorrhagic pattern may not be as useful for the determination of perfusion status. In cases with immediate onset BRVO, even if the posterior pole is ischemic, minimal pathologic changes might cause a discrepancy in associations with retinal perfusion status. Likewise, such a discrepancy might be caused in cases with severe occlusion of the affected retinal vein, which may accompany a faint retinal hemorrhage, and severe retinal perfusion impairment. In addition, the areas with retinal hemorrhage are considered to be accompanied by not only retinal ischemia, but also other vascular pathologies.

In conclusion, despite these shortcomings, we evaluated retinal hemorrhagic patterns at the posterior pole in acute BRVO both qualitatively and quantitatively. In the quantitative evaluation, the method of parallelism was successfully applied to the analysis using color fundus photography, and was useful in the differentiation of macular perfusion status. Because of its noninvasive nature, widespread availability, and ease of their evaluation, hemorrhagic patterns on fundus photography may be good clinical markers to determine the retinal perfusion status of eyes with acute BRVO.

## Methods

### Patients

The current study was approved by the Institutional Review Board (IRB) of Kyoto University Graduate School of Medicine, Kyoto, Japan, and adhered to the tenets of the Declaration of Helsinki. The 63 consecutive patients (63 eyes) with acute BRVO included in the current prospective study were examined in the Department of Ophthalmology at Kyoto University Hospital between June 2013 and June 2015. After the study design and the risks and benefits of participation were thoroughly explained, written informed consent was obtained from each participant.

The inclusion criteria of this study were: (1) major BRVO involving the temporal sector and macular BRVO, with retinal hemorrhage and retinal edema involving the macula; (2) a symptom duration of less than 3 months prior to examination. The diagnosis of BRVO was based on fundus examinations and FA findings and determined by retinal specialists (YM, TM). In the current study, eyes with central retinal vein occlusion (CRVO) were excluded, but eyes with hemi-CRVO were included. Eyes with co-existing retinal vascular disease (*i.e.*, retinal artery occlusion, diabetic retinopathy, hypertensive retinopathy, and anterior ischemic optic neuropathy), and eyes with glaucoma, high myopia (a refractive error of less than -6 diopters or an axial length of more than 26.5 mm), a senile cataract that resulted in poor image quality, and a history of ocular surgery except for cataract surgery were also excluded from the current study.

### Sample size and power calculations

As a pilot study, we measured parallelism in 10 eyes with nonischemic macula and in 10 eyes with ischemic macula, which were 0.33 ± 0.13 and 0.20 ± 0.11, respectively. When statistical α error and β error values were set as 0.05, and 0.20, respectively, and when the presumed ratio of patients with ischemic macula to those with nonischemic macula was set as 0.75, the total calculated sample size was 33. Considering the exclusion of some patients, we finally included a number of patients approximately twice the total calculated sample size.

### Examinations

During the initial visit, each patient underwent a comprehensive ophthalmic examination, which included measurement of best-corrected visual acuity (VA) with a Landolt chart and an intraocular pressure determination. Fundus biomicroscopy with a non-contact lens, wide-field digital fundus photography (Optos 200Tx imaging system, Optos PLC, Dunfermline, United Kingdom), and an OCT examination (Spectralis HRA + OCT, Heidelberg Engineering, Heidelberg, Germany) were performed after pupil dilation.

All eligible eyes with acute BRVO exhibited extensive retinal hemorrhage in the affected retinal areas. In the current study, we qualitatively classified the hemorrhagic pattern at the posterior pole in a masked fashion by two independent graders, and a third grader judged the pattern to resolve disagreement. Each eye was classified as having a non-flame-shaped (dot or blot) hemorrhage when not presenting any flame-shaped hemorrhage, or with a flame-shaped hemorrhage when presenting a flame-shaped (mainly flame- or fan-shaped, and occasionally splinter-shaped) hemorrhage, regardless of the amount, within the affected retina at the posterior pole between the fovea and the affected arcade vessels (Fig. 3). The graders had no access to the patient’s VA data or FA imaging.

To assess retinal perfusion status, each patient underwent FA with the Optos 200Tx imaging system. The retinal perfusion status of each eye was determined on early-phase Optos FA images exhibiting no dye leakage, and was classified as the nonischemic type when the retinal capillary dropout (nonperfusion) at the posterior pole was less than 10 disc areas (DA), and as the ischemic type when the area was equal to or greater than 10 DA. Retinal perfusion status was estimated in a masked fashion by two independent graders, and a third grader judged the perfusion status to resolve disagreement. The graders did not see the late phases of the FA images and had no access to the patients’ VA data or color fundus photography.

On OCT images, foveal retinal thickness was defined as the mean distance between the vitreoretinal interface and retinal pigment epithelium within a central subfield of the Early Treatment Diabetic Retinopathy Study grid.

### Calculation of parallelism

Uji and associates recently reported a new method of “parallelism” to quantitatively evaluate foveal pathomorphologies of eyes with an epiretinal membrane^24^ or diabetic macular edema^25^. Parallelism can be calculated using line segments after simple filtering and thresholding of the original images, and reflects how parallel and linearly objects lie side-by-side^22^,^23^. In the current study, we applied parallelism to quantitatively analyze the retinal hemorrhagic pattern at the posterior pole in acute BRVO using color fundus photography.

At the central area between the fovea and the point where a vertical line through the fovea intersects the affected arcade vessels, we cropped a rectangular region of interest (ROI) measuring 200 pixels × 200 pixels that covered the area of the retinal hemorrhage, and selected the 8-bit grayscale image from the split green channel of the original (Fig. 3). Because the appearance of the retinal hemorrhage was considered to be affected by both the shape of the hemorrhage itself and the surrounding retinal nerve fibers in which the hemorrhage sparsely existed, we quantified these 2 morphologies within the ROI and averaged the values for further analyses.

When evaluating the morphological features of a flame-shaped retinal hemorrhage, that is, the retinal hemorrhage and hemorrhage-sparse retinal areas, they seemed to be arranged in parallel; we therefore calculated their parallelism. Parallelism was expected to show higher values in eyes with a flame-shaped retinal hemorrhage and lower values in eyes with a non-flame-shaped dot or blot retinal hemorrhage (Fig. 3). Parallelism was calculated using a modified version of the previously described method for OCT image analyses^24^,^25^. Briefly, skeletonized images (lines) were extracted from cropped Optos images by applying a 4–5 pixel band-pass filter using ImageJ software (Wayne Rasband, National Institutes of Health, Bethesda, MD, USA; available at http://rsb.info.nih.gov/ij/index.html) and the Kbi_BandPass plug-in (http://hasezawa.ib.k.u-tokyo.ac.jp/zp/Kbi/ImageJKbiPlugins) followed by binarization with intensity thresholding using Otsu’s thresholding method for automatic binarization level decisions using the Kbi_ThrOtsu plug-in (http://hasezawa.ib.k.u-tokyo.ac.jp/zp/Kbi/ImageJKbiPlugins). The parallelism, which reflects the orientation of the line segments in the ROI, was then calculated by the following formula:


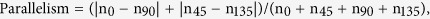


where n_0_, n_45_, n_90_, and n_135_ are the numbers of neighboring pixel pairs at 0°, 45°, 90°, and 135° with respect to the horizon, respectively^24^,^25^. Parallelism can range from 0 to 1, and images with more parallel line segments have higher parallelism.

### Statistical analysis

The statistical analysis was performed using PASW Statistics version 18.0 (SPSS, Chicago, IL, USA). All values are presented as the mean ± standard deviation. For statistical analysis, VA as measured with a Landolt chart was converted to the logarithm of the minimum angle of resolution (logMAR). Comparisons between the 2 groups were performed using the unpaired *t*-test, and differences in distributions were analyzed via chi-squared tests. Bivariate relationships between retinal perfusion status and other clinical factors were determined by computing the area under the receiver operating characteristic curve (AUROC), and sensitivities at fixed specificities were calculated. The AUROCs were compared using MedCalc version 12 (MedCalc Software, Ostend, Belgium). The cutoffs were calculated with MedCalc as the points with the best balances of sensitivity and specificity. Sensitivity at fixed specificities of 90% and 95% (10% and 5% false-positive rates, respectively) and positive and negative likelihood ratios were also calculated. A *P* value < 0.05 was considered statistically significant.

## Additional Information

**How to cite this article**: Muraoka, Y. *et al*. Association between retinal hemorrhagic pattern and macular perfusion status in eyes with acute branch retinal vein occlusion. *Sci. Rep.* **6**, 28554; doi: 10.1038/srep28554 (2016).

## Figures and Tables

**Table 1:** Characteristics of the Included Patients with Acute Branch Retinal Vein Occlusion.

Age (years)	71.8 ± 12.5
Sex (male/female)	23/40
Number of studied eyes	63/63
Macular branch retinal vein occlusion (eyes)	14
Major branch retinal vein occlusion (eyes)	40
Hemi-central retinal vein occlusion (eyes)	9
Duration of symptoms (months)	1.7 ± 1.3
LogMAR visual acuity	0.42 ± 0.36
Mean foveal thickness (μm)	564.8 ± 196.3
Retinal perfusion status at the posterior pole (nonischemic/ischemic/undeterminable)	30/28/5

All values are presented as the mean ± standard deviation; logMAR = logarithm of the minimum angle of resolution.

**Table 2:** Comparisons of Qualitative Ocular Features Associated with Retinal Perfusion Status in Eligible Eyes.

	Retinal perfusion status		*P*value
	non-ischemic (30 eyes)	ischemic (28 eyes)	
Cotton wool spots (eyes)	7	11	0.189
Retinal whitening (eyes)	0	2	0.136
Sheathed vessels (eyes)	1	5	0.069

LogMAR = logarithm of the minimum angle of resolution. Five eyes were excluded from the analysis because retinal perfusion status could not be determined due to blockage by a dense retinal hemorrhage.

**Table 3:** Comparisons of Clinical Measurements of Eyes with Acute Branch Retinal Vein Occlusion Divided by Macular Perfusion Status on the Affected Side.

	Retinal perfusion status at the posterior pole		*P*value
	Nonischemic (45 eyes)	Ischemic (13 eyes)	
Age (years)	71.2 ± 8.5	72.5 ± 16.0	0.693
Duration of symptoms (months)	1.5 ± 1.3	1.8 ± 1.3	0.294
LogMAR visual acuity	0.375 ± 0.321	0.464 ± 0.392	0.345
Mean foveal thickness (μm)	529.8 ± 163.7	602.3 ± 223.2	0.169
Retinal hemorrhagic pattern(flame-shaped/non-flame-shaped; eyes)	39/6	0/13	<0.001
Parallelism	0.362 ± 0.115	0.222 ± 0.126	<0.001

LogMAR = logarithm of the minimum angle of resolution. Five eyes were excluded from the analysis because retinal perfusion status could not be determined due to blockage by a dense retinal hemorrhage. Retinal perfusion status was determined in a central area between the fovea and the point where a vertical line through the fovea and the affected arcade vessels intersect.

**Table 4:** Area Under the Receiver Operating Characteristic Curve, Best Sensitivity-Specificity Balance, Likelihood Ratios, and Sensitivity at Fixed Specificities for Clinical Characteristics Differentiating Retinal Perfusion Status in Eyes with Acute Branch Retinal Vein Occlusion.

	AU ROC	95% CI	AU ROC *P*value	Cutoff points	Sensitivity (%)	Specificity (%)	+ LR	− LR	Sensitivity	
									90% Specificity	95% Specificity
Age (years)	0.537	0.393–0.676	0.730	45	0.00	78.57	0.00	1.27	0.00	0.00
Duration of symptoms (months)	0.515	0.375–0.654	0.851	2.7	71.79	6.67	0.77	4.23	15.38	12.82
LogMAR visual acuity	0.579	0.437–0.713	0.397	0.301	51.28	66.67	1.54	0.73	7.05	6.09
Mean foveal thickness (μm)	0.662	0.517–0.787	0.114	608	81.58	64.29	2.28	0.29	5.26	5.26
Parallelism (quantitative hemorrhagic pattern)	0.975	0.886–0.999	<0.001	0.185	94.59	100.00		0.05	97.30	94.59

AUROC = area under the receiver operating characteristic curve; CI = confidence interval; LR = likelihood ratio; LogMAR = logarithm of the minimum angle of resolution. Three eyes were excluded from this analysis because retinal perfusion status was undeterminable because of blockage by a dense retinal hemorrhage.
